# Burnout syndrome and job satisfaction in community health workers

**DOI:** 10.47626/1679-4435-2021-903

**Published:** 2024-02-16

**Authors:** Lorhan da Silva Menguer, Eduarda Valim Pereira, Antonio Carlos Rosa da Silva, Joni Marcio de Farias

**Affiliations:** 1 Educação Física, Universidade do Extremo Sul Catarinense (UNESC), Criciúma, SC, Brazil; 2 Grupo de Estudo e Pesquisa em Promoção da Saúde (GEPPS), UNESC, Criciúma, SC, Brazil; 3 Programa de Pós-Graduação em Saúde Coletiva, UNESC, Criciúma, SC, Brazil

**Keywords:** community health workers, occupational stress, job satisfaction, agentes comunitários de saúde, estresse ocupacional, satisfação no emprego

## Abstract

**Introduction:**

Community health workers play a prominent role in the primary care context in Brazil. Burnout syndrome is an important work-related condition whose consequences affect job satisfaction.

**Objectives:**

To evaluate the extent of burnout syndrome and job satisfaction among community health workers in a city in southern Santa Catarina.

**Methods:**

This analytical, individual, cross-sectional study was conducted in Family Health Units. The participants were selected through random probability sampling, with a sampling error of 10%. After sociodemographic data collection, the Maslach Burnout Inventory and a job satisfaction scale were applied.

**Results:**

All 66 included workers were women. According to the analyses, there was a moderate positive correlation between satisfaction with colleagues and satisfaction with supervisors. There were weak positive correlations between emotional exhaustion and depersonalization and between professional fulfillment and satisfaction with promotions. However, there was a weak negative correlation between emotional exhaustion and both professional fulfillment and satisfaction with salary

**Conclusions:**

The results indicate that the conditions of these workers are sufficiently satisfactory to deal with the demands of the job. Nevertheless, there was substantial dissatisfaction with salary, which can be a demotivator and trigger work-related depression.

## Abstract (pt)

**Introdução:**

Os agentes comunitários de saúde possuem um lugar de evidência na atual proposta da atenção básica. Uma das doenças no meio trabalhista é a síndrome de *burnout,* e, ao adquirir essa doença, o trabalhador sofre com consequências que afetam a satisfação no trabalho.

**Objetivos:**

Avaliar a existência da síndrome de *burnout* e a satisfação no trabalho dos agentes comunitários de saúde de uma cidade do extremo Sul catarinense.

**Métodos:**

Tratou-se de um estudo analítico, individual, transversal realizado nas Estratégias de Saúde da Família. Os participantes foram selecionados por meio de amostragem aleatória probabilística, com erro amostrai de 10%. Foi realizada anamnese de identificação, e os participantes responderam ao Inventário de *Burnout* de Maslach e à Escala de Satisfação no Trabalho.

**Resultados:**

Participaram do estudo 66 agentes comunitárias, todas do sexo feminino. As análises demonstraram correlação positiva moderada entre satisfação com os colegas e com o chefe, e correlações positivas fracas entre exaustão emocional e despersonalização, assim como entre realização profissional e satisfação com promoções. Por outro lado, exaustão emocional, realização profissional e satisfação com a remuneração obtiveram correlação negativa fraca.

**Conclusões:**

Os resultados indicam boas condições dos agentes comunitários para lidar com as demandas do trabalho. Destaca-se a insatisfação com a remuneração, podendo ser desmotivante e podendo desencadear doenças relacionadas à saúde mental.

## INTRODUCTION

Community health workers (CHW) play a very important role in Brazil's Unified Health System because they are the community representative of the health team, working in direct contact with the population in their homes, strengthening the ties between the community and primary health care services.^1^

In accordance with the National Basic Care Policy, CHW have specific responsibilities, including: (1) developing initiatives to integrate the health team and the population assigned to the health unit, (2) registering all residents in their micro-region and keeping the records updated, (3) informing families about the available health services, and (4) developing health promotion, disease (/aggravation) prevention, and health surveillance activities through home visits and individual and collective educational activities in homes and the health unit.^2^

These professionals often experience stressful situations because they reside where they work; they live according to community routines, which involves both positive and negative aspects.^3^ Burnout syndrome can be a major problem for these workers. This phenomenon results from continuous work-related stress, consisting of the following domains: emotional exhaustion, in which the energy required to perform work activities is depleted; depersonalization, which results in cold and impersonal behavior with colleagues and patients; and low professional fulfillment, in which professionals feel little fulfillment in their work activities.^4^

Various factors are associated with professional health care, including sociodemographic characteristics, low job satisfaction, and negative attitudes toward work, in addition to psychosocial factors, such as role ambiguity, inexperience, and the relationship with the health team.^4^ In southeastern Brazil, Vicente & Portes^5^ found a burnout prevalence of 34.72% among CHW, and a low but significant relationship with preventive care. Another study corroborated these findings, reporting that mental health issues related to CHW can be neither avoided nor modified, because they are inherent to the profession, although health education initiatives can raise awareness and outline strategies for dealing with these issues.^6^

Factors such as a high stress level, low job satisfaction, high turnover, high absenteeism, low productivity, and burnout (which affects physical health, mental health, and job performance and leads to a growing desire to quit) also affect the care provided to patients and their families.^7^ Understanding the relationships between workers and job satisfaction is a concern for researchers, since this relationship can influence the quality of life, affecting various aspects of health-related behavior.^8^

Assessing the health status of CHW can reflect their mental health and its reflection in practice. Such awareness can facilitate initiatives to reduce stress levels and job dissatisfaction, given that these professionals are, after all, responsible for health care and health promotion in the community.

Due to the importance of CHW in public health and the lack of studies on burnout syndrome and job satisfaction among these professionals, our objective was to determine job satisfaction levels and the prevalence of burnout syndrome, as well as the correlation of these factors.

## METHODS

### STUDY CHARACTERIZATION

This analytical, individual, cross-sectional study investigated the existence of burnout syndrome and job satisfaction among CHW in Criciúma, a city of in southern Santa Catarina, Brazil.

### STUDY LOCATION

The study was conducted in the Family Health Units of the following 5 health districts: Santa Luzia (10 units, 40 CHW), Rio Maina (8 units, 32 CHW), Boa Vista (8 units, 30 CHW), Centro (12 units, 53 CHW), and Próspera (10 units, 44 CHW).

### STUDY POPULATION

According to Municipal Health Department data from July 2019, 199 CHW were employed in the municipality's Family Health Units.

### SAMPLE

**Figure 1:**
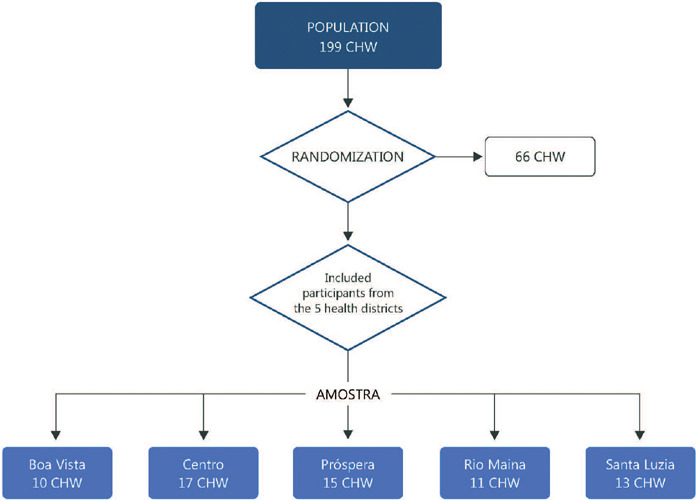
Sample flowchart, 2021 (n = 66). CHW: community health workers.

We used probabilistic random sampling, ie, all CHW had the same chance of being selected to participate. Of the 199 total CHW, 66 were randomly drawn (Figure 1). The inclusion criteria were active employment status as a CHW, availability to participate in the study, and providing written informed consent. The sample size was determined using Barbetta's formula, with a sample error of 10%.^9^

### DATA COLLECTION PROCEDURES

The study was designed in 3 stages. First, the project was approved by the Human Research Ethics Committee of the Universidade do Extremo Sul Catarinense (opinion 3,481,535) and was authorized by the Municipal Health Department. The manager of each health unit was then contacted by telephone to schedule a project presentation in the unit. The project was then presented to the CHW in each unit, at which point a lottery for study participants was held. The lottery procedures were the following: the Family Health Unit of each district was visited in alphabetical order of neighborhoods. One envelope was delivered to each CHW, randomly containing either an invitation to participate or a thank you note for attending the meeting. If any CHW who received an invitation chose not to participate, there would be a new raffle among the remaining CHW.

After the study was described to the CHW, all included volunteers provided written informed consent. The data collection was performed at the same meeting (between August and September 2019) by physical education professions from the Multiprofessional Residency Program in Public Health/Basic Care; the instruments were filled out together with a researcher.

### INSTRUMENTS

The participants provided sociodemographic data, including sex, education level, marital status, and age. This questionnaire was based on validated instruments. Weight (kg) was determined with a 200 kg capacity scale (Filizola SA, São Paulo, Brazil), and height (cm) was measured with a wall-mounted stadiometer. For the anthropometric measurements, the participants remained barefoot and with minimal clothing to ensure data reliability. Body mass index was then calculated as weight/height.^2^

Occupational stress was assessed with the Maslach Burnout Inventory. This self-applied instrument is answered on a 5-point Likert scale, varying from 1 (never) to 5 (always). It consists of 22 items in 3 independent dimensions: emotional exhaustion (9 items), depersonalization (5 items), and professional fulfillment (8 items).^10^ Scoring is based on the total points in each domain. Participants with high scores in the emotional exhaustion and depersonalization domains and low scores in the professional fulfillment domain were diagnosed with burnout syndrome.^11^ Emotional exhaustion scores between 0 and 15 points and depersonalization scores between 0 and 2 points are considered low or normal scores, while scores > 25 and > 8 for emotional exhaustion and depersonalization, respectively, are considered high. Professional fulfillment scores between 0 and 33 points are considered low.

#### Job satisfaction scale

The job satisfaction scale included 25 questions in 5 dimensions: co-workers, salary, supervisors, duties, and promotions. This self-applied instrument is answered on a 7-point Likert scale, varying from 1 (totally unsatisfied), 2 (very unsatisfied), 3 (unsatisfied), 4 (indifferent), 5 (satisfied), 6 (very satisfied), to 7 (totally satisfied).^12^

### DATA ANALYSIS

The data were input into a spreadsheet, represented by absolute values, mean (SD), and SEM. The data were initially analyzed descriptively to characterize sociodemographics, burnout, and job satisfaction. Data normality was assessed with the Kolmogorov-Smirnov test. Non-parametric analyses were used for non-normally distributed data. Student's *t*-test for independent samples was used to compare normally distributed means between groups. Other variables were analyzed with non-parametric Mann-Whitney and Wilcoxon U tests. Spearman's rank correlation coefficient was used to determine the relationship between burnout and job satisfaction. The statistical analysis was performed in IBM SPSS Statistics 22.0 (IBM, Armonk, NY, USA).

## RESULTS

### DESCRIPTIVE ANALYSES

All participants were women and the mean age was 43.8 (SD, 11.5) years. Their weight varied from 57.5 to 90.5 kg, with a mean of 74 (SD, 16.5) kg, while their mean height was 1.61 (SD, 6.4) cm. Thus, the mean body mass index was 28.7 (SD, 5.5) kg/ m2, indicating that they were generally overweight. A total of 59.1% were married, and 80.3% had completed secondary school or had incomplete higher education. The mean employment time as a CHW was 8.1 (SD, 16.9) years (Table 1).

**Table 1:** Characterization of community health agents, 2021 (n = 66)

	n	Mean	SD
Age (years)	66	43.8	11.5
Weight (kg)	66	74.0	16.5
Height (cm)	66	160.5	6.4
Body mass index (kg/m^2^)	66	28.7	5.5
Time employed (years)	66	8.1	16.9

	Frequency	Percentage	Cumulative percentage
Marital status			
Married	39	59.1	59.1
Single	14	21.2	80.3
Cohabiting	7	10.6	90.9
Divorced	4	6.1	97.0
Widowed	2	3.0	100.0
Total	66	100.0	
Education			
Complete primary/ incomplete secondary	1	1.5	1.5
Complete secondary/ incomplete tertiary	53	80.3	81.8
Complete tertiary	12	18.2	100.0
Total	66	100.0	

Occupational stress, assessed with the Maslach Burnout Inventory, is described in Table 2. The proportions of low, medium, and high emotional exhaustion levels were similar, with 39.4% having a low level. A total of in 54.5% had a low depersonalization score, 51.5% had a high professional fulfillment score, and 31.8% had a high emotional exhaustion score.

**Table 2:** Burnout classification with the Maslach Burnout Inventory, 2021 (n = 66)

	Frequency	Percentage	Cumulative percentage
EE			
Low	26	39.4	39.4
Meduim	19	28.8	68.2
High	21	31.8	100.0
DE			
Low	36	54.5	54.5
Meduim	19	28.8	83.3
High	11	16.7	100.0
PF			
Low	6	9.1	9.1
Meduim	26	39.4	48.5
High	34	51.5	100.0

DE = Depersonalization; EE = emotional exhaustion; PF = Professional fulfillment.

To characterize the job satisfaction scale (Table 3), the variables were rated, as established in the questionnaire, on a 7-point Likert scale (completely dissatisfied, very dissatisfied, dissatisfied, indifferent, satisfied, very satisfied, and completely satisfied) - items with a score of 0 were not included.

**Table 3:** Job satisfaction scale results, 2021 (n = 66)

	Frequency	Percentage	Cumulative Percentage
Coworkers			
Very unsatisfied	2	30	3.0
Unsatisfied	8	12.1	15.2
Indifferent	27	40.9	56.1
Satisfied	23	34.8	90.9
Very satisfied	6	9.1	100.0
Salary			
Completely unsatisfied	4	6.1	6.1
Very unsatisfied	9	13.6	19.7
Unsatisfied	25	37.9	57.6
Indifferent	20	30.3	87.9
Satisfied	8	12.1	100.0
Supervisors			
Completely unsatisfied	2	3.0	3.0
Very unsatisfied	2	3.0	6.0
Unsatisfied	5	7.6	13.6
Indifferent	16	24.2	37.9
Satisfied	28	42.4	80.3
Very satisfied	10	15.2	95.5
Completely satisfied	3	4.5	100.0
Overall job satisfaction			
Very Unsatisfied	1	1.5	1.5
Unsatisfied	8	12.1	13.6
Indifferent	31	47.0	60.6
Satisfied	24	36.4	97.0
Very satisfied	2	3.0	100.0
Promotions			
Completely unsatisfied	6	9.1	9.1
Very unsatisfied	7	10.6	19.7
Unsatisfied	19	28.8	48.5
Indifferent	31	47.0	95.5
Satisfied	3	4.5	100.0

Table 3 shows that the majority (40.9%) of CHAs were indifferent regarding their satisfaction with coworkers, while 34.8% were satisfied with coworkers. In relation to satisfaction with salary, 37.9% were unsatisfied. As for satisfaction with supervisors, 42.4% were satisfied.

The correlational analysis (Table 4) indicated a moderate positive correlation between satisfaction with colleagues and satisfaction with supervisors (0.56 p ≤ 0.001). Weak positive correlations were found between emotional exhaustion and depersonalization (0.46 p ≤ 0.001), professional fulfillment and job satisfaction (0.37 p ≤ 0.001), professional fulfillment and satisfaction with promotions (0.30 p ≤ 0.005), satisfaction with supervisors and job satisfaction (0. 40 p ≤ 0.001 *),* and overall job satisfaction and satisfaction with promotions (0.46 p ≤ 0.001).

**Table 4:** Correlational analysis, 2021 (n = 66)

	Emotional exhaustion	Professional achievement	Depersonalization	Satisfaction with colleagues	Satisfaction with salary	Satisfaction with superiors	Overall job satisfaction
Professional fulfillment	-0.322**						
Depersonalization	0.464**	-0.414**					
Satisfaction with colleagues	-0.174	-0.061	0.024				
Satisfaction with salary	-0.332**	0.151	-0.139	0.121			
Satisfaction with superiors	-0.097	0.082	0.089	0.560**	0.158		
Overall job satisfaction	-0.240	0.369**	-0.224	0.358**	0.297*	0.397**	
Satisfaction with promotions	-0.228	0.301*	-0.094	0.150	0.441**	0.282*	0.459**

* Correlation significant at the 0.05 level (bilateral).

** Correlation significant at the 0.01 level (bilateral).

Weak negative correlations were found between emotional exhaustion and professional fulfillment (0.32 p ≤ 0.001), emotional exhaustion and satisfaction with salary (0.33 p ≤ 0.001), professional fulfillment and depersonalization (0.41 p ≤ 0.001) (Table 4).

## DISCUSSION

Due to the prominent place of CHW in the current primary care context, this study determined and correlated the prevalence of burnout syndrome and job satisfaction level among these professionals, providing an important analysis to support health interventions for this population.

Burnout syndrome is a psychosocial disorder that affects workers exposed to chronic stress at work, including excessive workloads, emotional exhaustion, depersonalization, and low professional fulfillment.^11^ Although many participants (39.4%) had a low emotional exhaustion level, one-third of the sample had a high level. Tironi et al.^13^ reported that emotional exhaustion is the initial symptom of burnout to emerge from occupational stress. They noted that after the onset of emotional exhaustion, workers had difficulty relaxing, which leads to physical fatigue, impeding daily activities.

Most of our sample reported a low level of depersonalization. This result is satisfactory, since depersonalization is associated with an impersonal and dehumanized professional milieu. According to Selamu et al.,^4^ workers manifest depersonalization through cynical and sarcastic behavior towards others.

More than half (51.5%) of the sample had high professional fulfillment scores. One factor that contributes to professional fulfillment is job type. CHW feel useful in the community where they live and work. Studies on health professionals have shown high professional fulfillment levels.^14,15^ According to our results, the risk of burnout syndrome was not high among these professionals.

A total of 42.4% of the CHW were satisfied with their superiors, which corroborates other studies that have applied a job satisfaction scale to civil servants.^16,17^ Satisfaction with superiors involves issues of professionalism, the way information is conveyed, and the way tasks are assigned to subordinates.^12^ The overall job satisfaction results were also good, with 36.4% reporting they were satisfied with the nature of their work. To achieve such a level of satisfaction, workers must be fully involved in their tasks,^12^ and involvement is closely linked with the role of HW.

Indifference was the most frequent response regarding colleagues and promotions, which warrants special attention on an institutional level, since this indicates the need to review the assessment process.^16^ Most (37,9%) of the CHW expressed some degree of dissatisfaction with their salary, which aligns with the results of other studies.^11,18^ Dissatisfaction with salary is a critical issue in the literature, highlighting the close relationship between salary and job satisfaction.^19^

We found a moderate positive association between satisfaction with supervisors and satisfaction with colleagues. Mutual support at work, ie, joint interaction between co-workers and supervisors to perform necessary tasks, can reduce worker stress and lower health risks.^20^

Emotional exhaustion was negatively associated with professional fulfillment and salary. It is worth noting that a lack of energy, cynicism, stress, frustration, and tension are linked to EE.^4^ Work overload and social conflict reduce the amount of time workers spend at work and the effort they expend on it.^21^

The present study was carefully designed to avoid limitations regarding its objectives, including a well-designed methodology, potential inclusion of the entire available population, and data collection by a trained team. Nevertheless, even though data collection was anonymous, some participants may have felt insecure about revealing their true feelings, which could have affected the results.

Although the job satisfaction rate was positive, with few cases of burnout, the importance of initiatives for this population cannot be dismissed. Thus, for future studies, we recommend extension activities aimed at caring for the caregiver, which could be developed in partnership with universities and the municipal health system. Such studies could involve health education interventions to encourage these professionals to take even better care of their own health so they can better care for the local population and deal with the stress of their profession.

## CONCLUSIONS

This study characterized the occupational stress and job satisfaction profile of CHW in a city in southern Brazil. Our results were positive, indicating that the workers had good overall conditions and could deal with the demands of work without excessive emotional overload. Nevertheless, some participants had poor results, which is concerning and indicates a context of relative vulnerability to burnout. This highlights the importance of preventive interventions to avoid increasing this risk. It is also important to point out that our participants' profile of may not apply to CHW in other cities/regions. Investing in the health and quality of life of health care providers ensures better health for the entire community.
